# Genetic polymorphisms of *RANTES, IL1-A, MCP-1 *and *TNF-A *genes in patients with prostate cancer

**DOI:** 10.1186/1471-2407-8-382

**Published:** 2008-12-19

**Authors:** Pablo Sáenz-López, Rafael Carretero, José Manuel Cózar, José Maria Romero, Julia Canton, José Ramón Vilchez, Miguel Tallada, Federico Garrido, Francisco Ruiz-Cabello

**Affiliations:** 1 Servicio de Análisis Clínicos e Inmunología, Hospital Universitario Virgen de las Nieves, Granada, Spain; 2 Servicio de Urología, Hospital Universitario Virgen de las Nieves, Granada, Spain

## Abstract

**Background:**

Inflammation has been implicated as an etiological factor in several human cancers, including prostate cancer. Allelic variants of the genes involved in inflammatory pathways are logical candidates as genetic determinants of prostate cancer risk. The purpose of this study was to investigate whether single nucleotide polymorphisms of genes that lead to increased levels of pro-inflammatory cytokines and chemokines are associated with an increased prostate cancer risk.

**Methods:**

A case-control study design was used to test the association between prostate cancer risk and the polymorphisms *TNF-A*-308 A/G (rs 1800629), *RANTES*-403 G/A (rs 2107538), *IL1-A*-889 C/T (rs 1800587) and *MCP-1 *2518 G/A (rs 1024611) in 296 patients diagnosed with prostate cancer and in 311 healthy controls from the same area.

**Results:**

Diagnosis of prostate cancer was significantly associated with *TNF-A *GA + AA genotype (OR, 1.61; 95% CI, 1.09–2.64) and *RANTES *GA + AA genotype (OR, 1.44; 95% CI, 1.09–2.38). A alleles in *TNF-A *and *RANTES *influenced prostate cancer susceptibility and acted independently of each other in these subjects. No epistatic effect was found for the combination of different polymorphisms studied. Finally, no overall association was found between prostate cancer risk and *IL1-A *or *MCP-1 *polymorphisms.

**Conclusion:**

Our results and previously published findings on genes associated with innate immunity support the hypothesis that polymorphisms in proinflammatory genes may be important in prostate cancer development.

## Background

Chronic or recurrent inflammation is known to play a causative role in the promotion and progression of many human tumours, including cancers of the liver, oesophagus, stomach, large intestine and urinary bladder [1-3]. Chronic inflammation has also been implicated in the aetiology of prostate cancer [4-7]. Prostate cancer risk has been associated with sexually transmitted infections and prostatitis in some epidemiologic studies [8,9], and its relationship with genetic polymorphisms in inflammatory cytokines has been explored in various case-control studies [10-13]. Chronic inflammation, alongside the intrinsic properties of pre-malignant cells and other determinants, may therefore be one of the driving forces of malignant transformation. Thus, numerous mediators released in dysregulated chronic inflammation have been found to promote cell growth and invasion, induce mutagenesis and increase angiogenicity [1]. By virtue of these properties, inflammatory mediators favour initiation of malignancy, and if sustained, may also promote progression. In general, many inflammatory conditions are characterized by the recruitment of inflammatory cells, predominantly macrophages, due to the wide variety of bioactive products they release. Cytokines and chemokines produced by activated innate immune cells are the most important components orchestrating the inflammatory-tumour-microenviroment [14]. Most studies have focussed on inflammatory chemokines that promote monocyte migration, primarily MCP-1 and also RANTES (regulated upon activation, normally T cell-expressed and presumably secreted) [15-17]. Full activation of NF-κB in inflammatory leukocytes resident in preneoplastic sites are likely to exacerbate local M1-inflammation (high TNF-A, high IL-1, high IL-12, low IL-10, low TGF-β) and favour tumourigenesis [18]. In particular, pro-inflammatory cytokines (e.g. IL-1 and TNF), can themselves affect cancer risk [1,5,8,19].

In the present investigation, we studied the role of several polymorphisms reported to have functional and biological relevance in the inflammatory process. The SNPs selected for study have all been found to influence the expression of their respective cytokine in vitro.

TNF is a key mediator of inflammation and may contribute to tumour initiation by stimulating production of genotoxic molecules, which can lead to DNA damage and mutations [20]. Inter-individual variations in TNF-A levels have been attributed to polymorphisms, notably the A allele at -308 (G/A) position in the promotor region of the *TNF *gene, which has been associated with higher *TNF-A *transcription levels [21] and an increased risk of several types of cancer [22]. However, findings on *TNF-A *polymorphisms in prostate cancer have been inconsistent [10-13]. In fact, evaluation of genetic variation in cytokines and chemokines in relation to inflammation and cancer risk is a complex task. Thus, it is important to investigate the possible role of other genes involved in the inflammation pathway that might influence prostate cancer risk.

Proinflammatory cytokines IL-1A and IL-1B [23,24] are also produced by monocytes, macrophages and epithelial cells and exhibit similar biological characteristics to TNF-A, including participation in host response to microbial invasion, inflammation and tissue injury [20]. *IL-1 *polymorphisms have also been linked to gastric [25], hepatocellular [26], lung [27], colorectal [28], vulvar [29] and ovarian [30] cancers. Allele T of the *IL1 (-*889 C>T) polymorphism has been associated with high production of this cytokine [31].

RANTES chemokine is believed to play a role in antitumour immunity *via *immune cell recruitment [32]. Polymorphisms in *RANTES *have been associated with a higher risk of pancreatic cancer, supporting the hypothesis that proinflammatory gene polymorphisms, in combination with proinflammatory conditions, may influence the development of pancreatic cancer [33].

Finally, MCP-1, a member of the CC chemokine family, possesses chemotactic activity for monocytes and T lymphocytes [34,35] and has been proposed to play a key role in macrophage recruitment, expression of angiogenic factors and activation of matrix metalloproteinases in breast cancer patients. Genetic variants within regulatory regions of *MCP-1 *that affect transcription and protein production have been correlated with the risk of breast cancer metastasis [36]. This finding is consistent with the previously reported overexpression of *MCP-1 *in breast cancer tissue [37].

These cytokines and chemokines were selected for our study because they are released in response to various forms of cellular stress, including inflammation and carcinogen exposure. The objective of this study was to assess the influence of genetic polymorphism of *RANTES, MCP-1, IL-1A *and *TNF-A *on the risk of prostate cancer.

## Methods

### Patient samples

The study included adult males with a recent diagnosis of prostate cancer (n = 296) referred to our hospital between 2002 and 2007. Clinical diagnosis of primary adenocarcinoma of the prostate was histopathologically confirmed after abnormal serum PSA findings and/or lower urinary tract symptoms. Patients were unrelated Caucasian men with a mean age of 67.4 ± 5.8 yrs and mean PSA level of 31.6 ± 5.7 (Table 1). Healthy unrelated Caucasian men (n = 311) were enrolled as controls; these were recruited from among blood donors on the Spanish Bone Marrow Donor Registry of the same geographic region and with no history of prostate cancer according to Registry records. The mean of age of the controls was 44 ± 11 yrs. The clinical characteristics of study participants are presented in Table 1. The patients were classified according to their disease progression, Gleason score and PSA level. Peripheral blood samples were drawn from all participants into tubes containing K3-EDTA. All subjects were interviewed by a researcher and signed their informed consent to participation in the study, which was approved by the Ethics Committee of our hospital.

**Table 1:** Clinical caracteristics of prostate cancer patients.

	**Case Subjects No**.	**(%)**		**Case Subjects No**.	**(%)**
Variables			Variables		

**Age at study**			**Gleason Score**		

<60	51		<4	15	5

60–69	140		4–6	122	41.2

70–79	89		7–8	133	45

>80	16		9–10	26	8.8

**T Stage**			**Differential Grade**		

T1	25	8.5	1	25	8.5

T2	112	37.8	2	245	82.7

T3	111	37.5	3	26	8.8

T4	48	16.2	**PSA levels, ng/ml**		

**N Stage**			<10	69	23.3

N0	187	63.2	10–19.99	68	23

N1	109	36.8	20–20.99	39	13.2

**M Stage**			30–30.99	20	6.7

M0	226	76.3	40–40.99	18	6

M1	70	23.7	50–50.99	22	7.4

			60–99.99	39	13.2

			>100	21	7

### DNA samples and genotyping

Total genomic DNA was isolated from peripheral blood of prostate cancer patients and healthy controls using the QIAamp DNA Mini kit. SNPs samples were genotyped by means of a Taqman 5' allelic discrimination assay. The PCR and genotype of each sample was automatically attributed by measuring the allelic specific fluorescence on 500 PCR-REAL TIME Sequence Detection System using SDS 2.2.1 software for allelic discrimination (Applied Biosystems, Foster City, CA, USA). Single nucleotide polymorphism amplification assays were used according to the manufacturer's instructions. Briefly, 10 ng of sample DNA(1 μl) were placed in 4 μl of reaction solution containing: 2.5 μl of the 2× TaqMan^® ^Universal PCR Mix (Applied Biosystems), 0.25 μl of predevel ped assay reagent from the SNP genotyping product(20×) (Applied Biosystems) containing two primers and two MGB-Taqman probes, and 1.25 μl of distilled water. Reaction conditions of the PCR were: preincubation at 50°C for 2 min and at 95°C for 10 min, followed by 40 cycles of 95°C, 15 s; 60°C, 1 min. The polymorphisms studied were: *TNF*-308 A/G (rs 1800629) SNP, *RANTES*-403 G/A (rs 2107538) SNP, *IL-1A*-889 C/T (rs 1800587) SNP, and *MCP-1 *2518 G/A (rs 1024611) SNP. We studied the polymorphism in the promoter region that affects its transcriptions. Thus, in *RANTES*, polymorphism A/G in region -403 of the promoter was studied; the A allele is associated with higher transcription [38]. In *MCP-1*, polymorphism A/G in region -2518 of the promoter was studied; the G allele produces much higher *MCP-1 *expression [39]. In *IL1-A*, polymorphism C/T in region -889 of the promoter was studied; the T allele causes higher *IL1-A *expression[31]. In *TNF-A*, polymorphism G/A of region -308 of the promoter was studied; the A allele produces higher *TNF-A *expression [40].

### Statistical analyses

Data were compiled according to the genotype and allele frequencies estimated from the observed number of each specific allele. SNPs allele frequencies were tested against Hardy-Weinberg equilibrium by comparing observed with expected genotype frequencies using a χ^2 ^test. The present study has a power higher than 95% to detect an Odds Ratio (OR) of 2 in all analyses (measured by Episheet programme). Genotype frequencies were compared in 2 × 2 tables with the χ^2 ^test. The strength of association was estimated by calculating ORs with 95% CI and p values. Binary logistic regression analysis was used to confirm differences in SNP frequencies between cases and controls. Interaction analysis was calculated by logistic binary regression. The SPSS v.15.0 software package was used for the statistical analyses.

## Results

The study included 296 patients recently diagnosed with prostate cancer and 311 blood donors (controls). Table 2 shows the genotype and allelic distributions of the polymorphisms in cases and controls, with estimated ORs. None of the SNP genotype frequencies deviated from Hardy-Weinberg equilibrium, and the distributions of alleles in the control group were in agreement with findings in other Spanish Caucasian populations [41]. Polymorphisms could sometimes not be genotyped because of PCR amplification problems, explaining discrepancies in the numbers of cases among groups. Associations were obtained by comparing with the control population (not age-matched). Comparison of *TNF-A *promoter polymorphisms between patients and controls revealed statistically significant differences in genotype distribution for locus -308, with a higher frequency of A carriers (AA/AG) in patients than in controls (OR = 1.61; 95% CI: 1.09–2.64 *p *= *0.017*). Patients also showed a higher frequency of A allele in the *RANTES *-403 G/A polymorphism versus controls (OR = 1.44; 95% CI: 1.09–2.38 *p *= *0.039*). In contrast, *MCP-1 *2518 G/A and *IL-1 A*- 889 T allele genotypes showed no significant differences between prostate cancer and control groups (Table 2), suggesting that these two genotypes may not play a role in susceptibility to prostate cancer.

**Table 2:** Statistical analysis of allele and genotype distributions of *IL1A, MCP-1, RANTES *and *TNF-A *polymorphisms.

**Genotype frequencies**	**Control** **n = 311**		**Case** **n = 298**		**Genotype results**	**p value**	**OR**	**95% confidence interval**	**Interaction análysis**
						
	**n**	**%**	**n**	**%**					
*Il-1 CC*	152	50.2	133	44.9	Il-1 CT/TT versus CC [25]	0.200	1.23	0.89 – 1.82	
					
*Il-1 CT/TT*	151	49.8	163	55.1					

*TNF-alpha GG*	256	82.6	221	74.7	TNF-alpha AG/AA versus GG [13]	0.017	1.61	1.09 – 2.64	
					
*TNF-alpha AG/AA*	54	17.4	75	25.3					

*MCP-1 AA*	178	57.2	174	58.4	MCP-1 AG/GG versus AA [27]	0.770	0.95	0.66 – 1.33	
					
*MCP-1 AG/GG*	133	42.8	124	41.6					

*RANTES GG*	221	72.5	192	64.6	RANTES AG/AA versus GG [16]	0.039	1.44	1.09 – 2.38	
					
*RANTES AG/AA*	84	27.5	105	35.4					

					TNF-alpha AG/AA and RANTES AG/AA	0.012	2.45	1.22 – 4.94	0.416

**Allele frequencies**					**Allele results**				

*Il-1 C*	426	70.3	401	67.5	Il-1 C versus Il-1 T	0.297	1.14	0.89–1.46	
					
*Il-1 T*	180	29.7	193	32.5					

*TNF-alpha G*	564	90.97	512	86.5	TNF-alpha G versus A	0.013	1.57	1.10–2.26	
					
*TNF-alpha A*	56	9.03	80	13.5					

*MCP-1 A*	479	77.01	448	75.2	MCP-1 A versus G	0.451	1.11	0.85–1.44	
					
*MCP-1 G*	143	22.99	148	24.8					

*RANTES G*	522	85.57	478	80.5	RANTES G versus A	0.018	1.44	1.03–1.95	
					
*RANTES A*	88	14.43	116	19.5					

We stratified the data, grouping subjects according to their carrier status for *IL1-A *allele T, *TNF-A *allele A, *RANTES *allele A and *MCP-1 *allele G. In logistic regression analyses, no significant association with prostate cancer risk was found for *IL1 *or *MCP-1 *allele carrier status, but subjects with AG or AA genotypes for *TNF-A *or *RANTES *had increased prostate cancer risk versus those with GG genotype (Table 2). The combination of the A allele of *TNF *and *RANTES *markedly increased this risk (OR, 2.45; 95% CI, 1.22 – 4.94, *p *= *0.012*), although no epistatic effect (*p *= *0.416*) was observed between these polymorphisms. SNP genotypes were also compared within the patient group and stratified according to tumour stage and serum PSA level (Table 1) to determine whether the genotypes influenced these indicators. No significant associations were observed.

## Discussion

Several polymorphisms have been associated with the severity of or susceptibility to numerous inflammatory diseases. In this case-control study, we investigated the association between polymorphisms in cytokine/chemokine genes (*TNF-A *-308 G/A, *RANTES*-403 G/A, *IL-1A *-889 C/T and *MCP-1 *2518 G/A) and prostate cancer risk. Reports on *TNF-A *gene polymorphisms and prostate cancer have been controversial [10-13]. Our results suggest that the A allele, associated with a high production of TNF-A, is linked to an increased risk for prostate cancer. TNF-A, a major factor in the inflammation response, has been related to many types of cancer, e.g., T-cell large granular lymphocyte leukaemia [42], cholestatic liver cancer, multiple myeloma, bladder cancer, hepatocellular carcinoma, gastric cancer and breast cancer [22]. However, no association has been described with nasopharyngeal carcinoma [43] or cervix carcinoma [44]. Discrepancies in some previously published results for *TNF-A *gene polymorphisms in prostate cancer may be related to the modest risk found. Inflammation is a highly complex process that involves hundreds of genes. The fact that numerous gene polymorphisms contribute to the inflammatory response adds further complexity to the analysis of a specific polymorphism, because each individual gene is likely to contribute only modestly to the risk. Thus, TNF-A may interact in important ways with multiple cytokine/chemokines involved in inflammation pathways that also display genetic variation.

The discrepant results may be explained by the effects of genetic heterogeneity and different gene-environment interactions. Although the distribution of -308A *TNF-A *has been found to vary among control groups due to ethnic variations [44], the distribution of the allelic frequencies in our study was similar to a recent report [13]. The influence of other (non-inflammation-related) genes or environmental factors is more likely to account for differences. Thus, there is evidence that prostate cancer has a complex and multifactorial aetiology, which might explain discrepancies in the attribution of prostate cancer risk to a single SNP [45]. Furthermore, discrepant results between the NorthAmerican [13] and the present study might be influenced by differences in diet, lifestyle and NSAID use between the study populations.

In this context, differences in *NF-kB *polymorphism were reported between Spanish and North American groups in association with ulcerative colitis [46]. Finally, a Sweden Study [47,48] found that increased risk for this type of tumour is probably also influenced by other genes that regulate inflammation (Toll-like receptor) and modify individual susceptibility to prostate cancer. The combined effect of several polymorphism loci is likely to markedly increase the risk of prostate cancer.

Gene-cluster polymorphisms in the *interleukin-1 *(*IL1B*) are associated with an increased risk of gastric and other types of cancer [25,30,49]. However, no associations have been found between polymorphisms in the IL1 gene cluster (*IL1A, IL1B *and *IL1R*) and ovarian or breast cancer [50,51]. Other authors reported no overall association between IL-1 polymorphisms and gastric cancer [52], and the reason for these different findings are unclear. The present case-control study in Caucasian men found no significant effect of *IL1A *(-889 C>T) gene polymorphisms on prostate cancer risk.

Chemokines are also expressed in inflammation, attracting and recruiting populations of immune effector cells to injury or infection sites, and the relationship between prostate cancer risk and polymorphisms of *RANTES *and *MCP-1 *was investigated in this study. No link with the *MCP-1 *polymorphism was observed, but a significant association was found between the A allele of *RANTES *and prostate cancer risk (*p *= * 0.039*). Allele A in *RANTES *is associated with increased transcription, which is related to inflammation and antitumour immunity *via *immune cell recruitment [53]. The allele A of *RANTES *and *TNF-A *has been associated with pancreatitis, especially as a possible early sign of pancreatic cancer [33]. In our study, the same alleles (*TNF-A *and *RANTES*) were also associated with a higher prostate cancer risk. Interestingly, the simultaneous presence of allele A of both genes promotes a higher risk (OR = 2.45, *p *= *0.012*) of cancer, but the interaction analysis showed no epistatic interaction between them (*p *= * 0.416*), indicating that their effects were additive. Finally, our study has some evident limitations and false positive results cannot be ruled out: thus, size of the study population is relatively small, aged-matched controls were not used and a possible bias corresponding to age cannot be excluded. However, even if we would have used a totally age-matched control subjects, our results would have been with even higher significance, because in this control group we cannot rule out a possibility that some of the younger control subjects may develop prostate cancer later in their life. In addition, there was no difference found between our control subjects and those used from our geographic area in a study of age-related diseases [54,55]. In general, we would like to comment that the influence of age on the immune gene polymorphism remains controversial. Some publications indicate that there are no differences in genotype distribution between older and younger controls [56], whereas others have reported an age-dependent difference in polymorphism of certain genes [57].

There is a need for studies of larger patient groups, including other potential functional polymorphisms in linkage disequilibrium to determine the role of these polymorphisms in prostate cancer.

## Conclusion

In conclusion, our data suggest an important role for *TNF-A *and *RANTES *polymorphisms in prostate cancer, as observed in many other types of cancer. Other genetic polymorphisms involved in innate immunity and chronic inflammation have been associated with prostate cancer [48], and the present findings support the hypothesis that the inflammatory process may constitute an important step in the initiation and promotion of prostate cancer.

## Competing interests

The authors declare that they have no competing interests.

## Authors' contributions

PSL, JC and RC performed analyses, managed the storage of blood samples and performed genotyping. JMR and JRV performed statistical analyses and programming. JMC and MT were responsible for data collection and manuscript editing. FG and FRC conceived of the study, participated in its design and coordination and drafted the manuscript. All authors have read and approved the final manuscript

## Pre-publication history

The pre-publication history for this paper can be accessed here:


